# Supplementary material for: Genome editing of phylogenetically distinct bacteria using portable retron-mediated recombineering
Source: bioRxiv. 2025 Jul 9:2025.06.16.660010. Preprint. [Version 2] doi: 10.1101/2025.06.16.660010 (PMC12262390; doi:10.1101/2025.06.16.660010)
Supplement: 2 [file NIHPP2025.06.16.660010v2-supplement-2.pdf]

bioRxiv preprint doi: https://doi.org/10.1101/2025.06.16.660010; this version posted July 9, 2025. The copyright holder for this preprint (which was not certified by peer review) is the author/funder, who has granted bioRxiv a license to display the preprint in perpetuity. It is made available under aCC-BY 4.0 International license.

SUPPLEMENTARY FIGURES

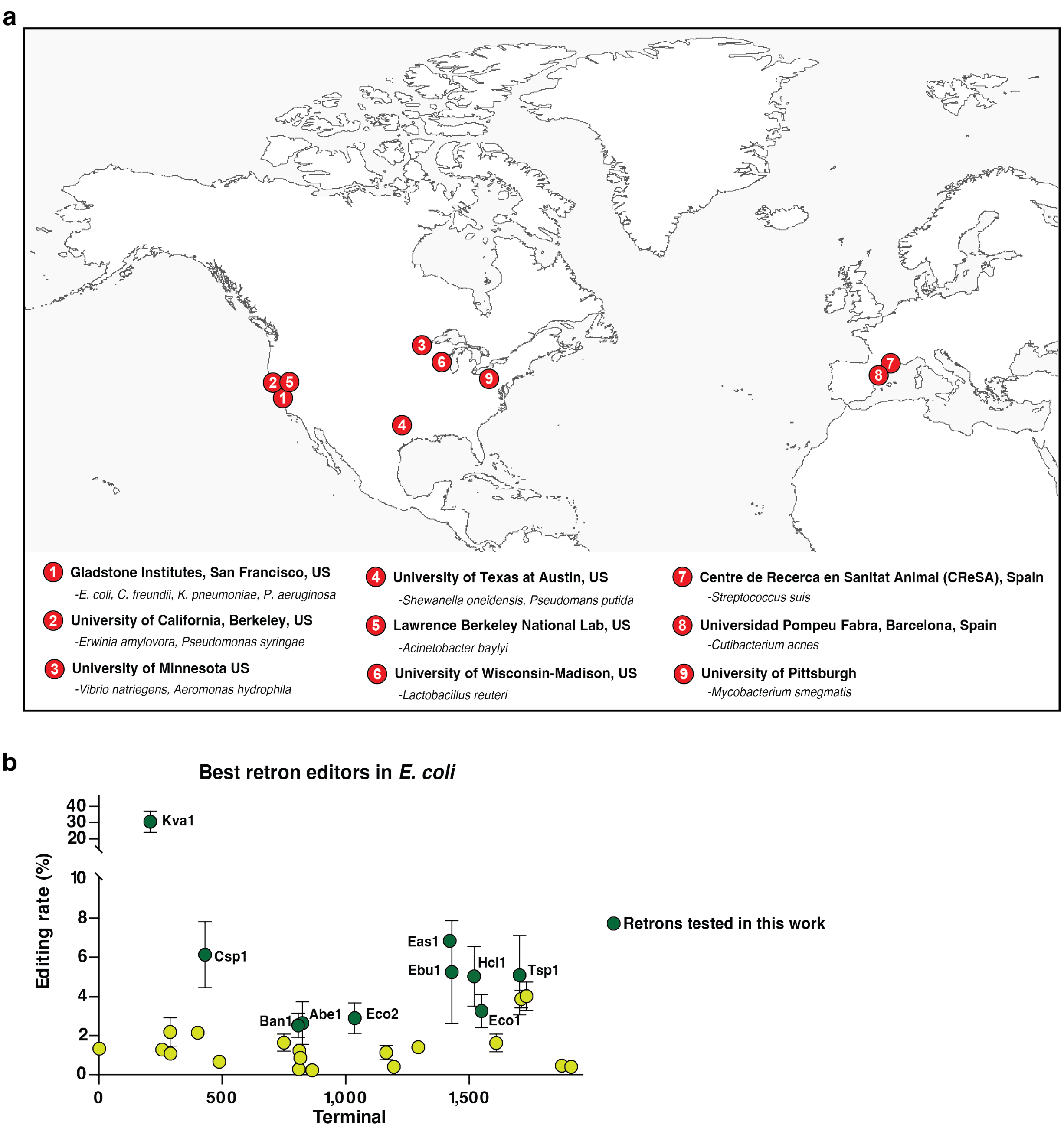

Supplementary Figure 1 – Geographic distribution of the research lab involved in the recombitron portability project and previous performance of the retrons used in this survey. a, Map showing the Academic Institutions involved in this project and the species assayed in each one of the research labs. **b**, Precise editing rate across retrons for bacterial genome recombineering. Points show mean ± standard error of the mean for three biological replicates. The name of the 10 selected retrons is indicated. Adapted from Khan et al., 2024.

bioRxiv preprint doi: https://doi.org/10.1101/2025.06.16.660010; this version posted July 9, 2025. The copyright holder for this preprint (which was not certified by peer review) is the author/funder, who has granted bioRxiv a license to display the preprint in perpetuity. It is made available under aCC-BY 4.0 International license.

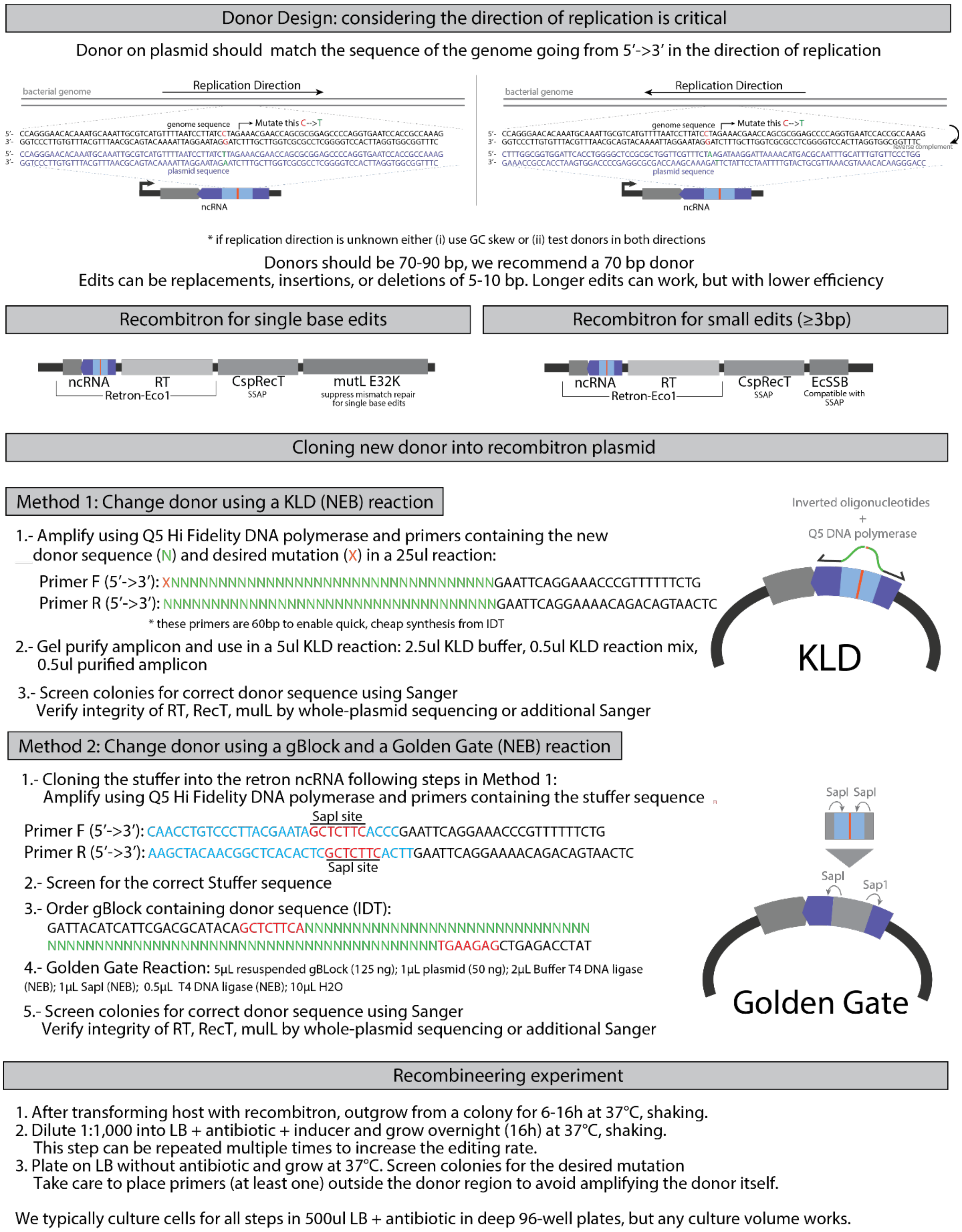

Supplementary Figure 2 – Protocol to design, construct and assay a recombitron for genome editing.

bioRxiv preprint doi: https://doi.org/10.1101/2025.06.16.660010; this version posted July 9, 2025. The copyright holder for this preprint (which was not certified by peer review) is the author/funder, who has granted bioRxiv a license to display the preprint in perpetuity. It is made available under aCC-BY 4.0 International license.

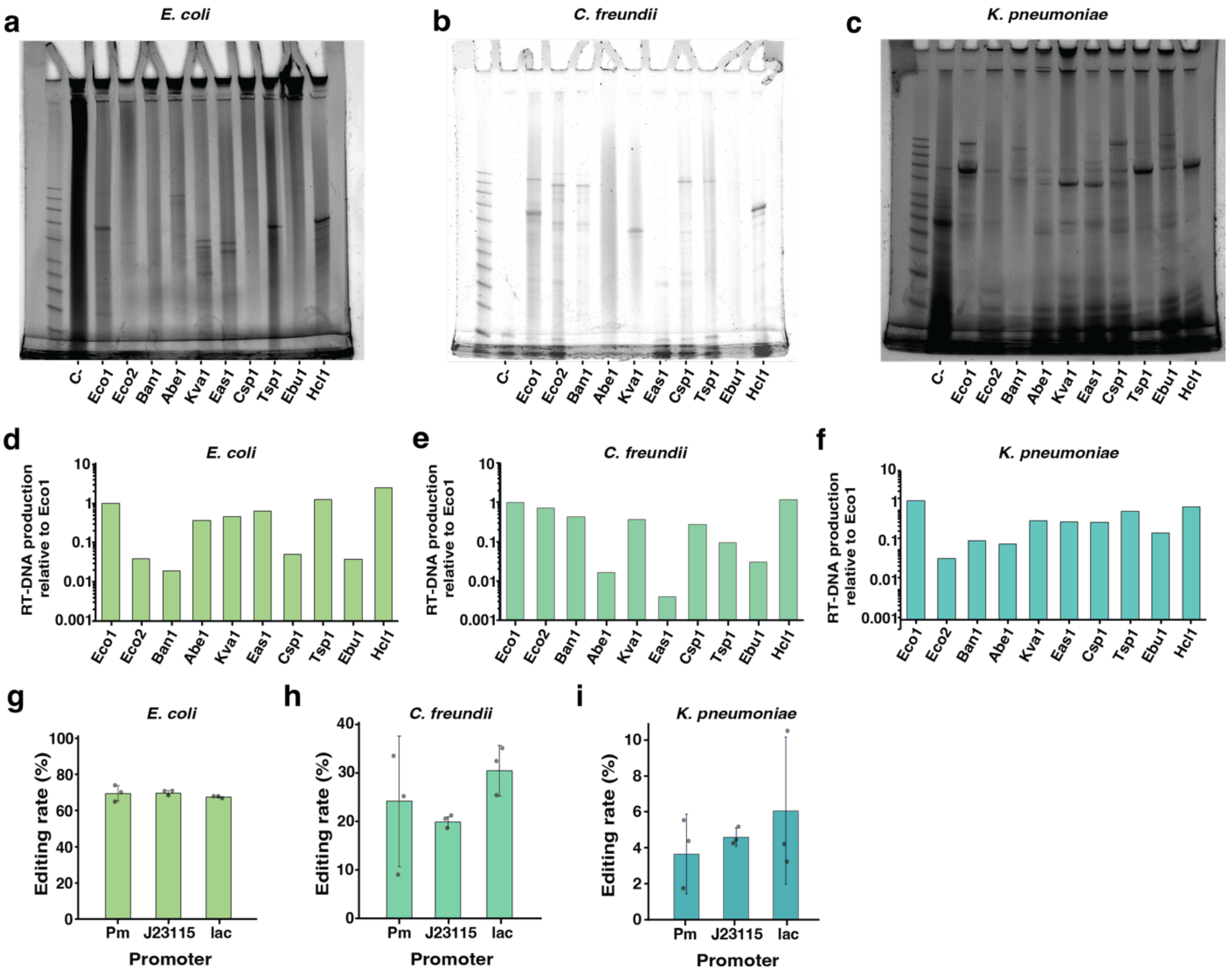

Supplementary Figure 3 – Retron-mediated recombineering in coliforms. **a**, Uncropped gel from **Fig 2b**, showing RT-DNA production from the set of 10 recombitrons in *E. coli*. **b**, Uncropped gel from **Fig 2c**, showing RT-DNA production from the set of 10 recombitrons in *C. freundii*. **c**, Uncropped gel from **Fig 2d**, showing RT-DNA production from the set of 10 recombitrons in *K. pneumoniae*. **d**, Quantification of RT-DNA production by density, relative to retron-Eco1 in *E. coli*. **e**, Quantification of RT-DNA production by density, relative to retron-Eco1 in *C. freundii.* **f**, Quantification of RT-DNA production by density, relative to retron-Eco1 in *K. pneumoniae*. In **d-f**, Density of the band produced by each retron was quantified with ImageJ software. **g**, Quantification of precise genome editing to make a 5 bp deletion in the *lacZ* gene in *E. coli* using Pm, lac or J23115 promoter. **h**, Quantification of precise genome editing to make a 5 bp deletion in the *lacZ* gene in *C. freundii* using Pm, lac or J23115 promoter. **i**, Quantification of precise genome editing to make a 5 bp deletion in the *lacZ* gene in *K. pneumoniae* using Pm, lac or J23115 promoter. In **g-i**, data were quantified by sequencing after 24 h of editing using Illumina NextSeq, circles show each of the three biological replicates, and errors bars are mean ± standard deviation.

bioRxiv preprint doi: https://doi.org/10.1101/2025.06.16.660010; this version posted July 9, 2025. The copyright holder for this preprint (which was not certified by peer review) is the author/funder, who has granted bioRxiv a license to display the preprint in perpetuity. It is made available under aCC-BY 4.0 International license.

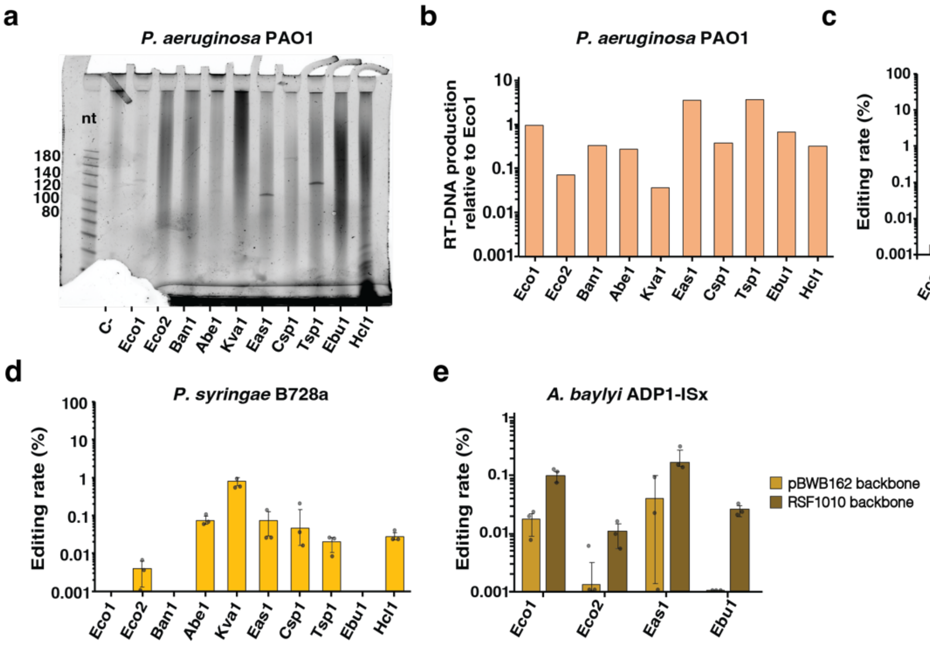

Supplementary Figure 4 – Retron-mediated recombineering in *Pseudomonadales*. a, PAGE analysis of RT-DNA production in *P. aeruginosa* PAO1. **b**, Quantification of RT-DNA production by density, relative to retron-Eco1 in *P. aeruginosa.* Density of the band produced by each retron was quantified with ImageJ software. **c**, Quantification of precise genome editing to make a 4 bp deletion targeting the *phzM* gene in *P. aeruginosa* PAO1 using the 10 recombitrons set with pBBR1 inverted origin of replication. **d**, Quantification of precise genome editing to make a 5 bp deletion in a non-essential intergenic region in *P. syringae* B728a using the 10 recombitrons set with pBBR1 inverted origin of replication. **e**, Comparison of precise genome editing to make a 4 bp deletion targeting a glycosyltransferase gene in *A. baylyi* sFAB6437 using recombitrons expressed from pWBW162 or RSF1010 backbone. In **c-e**, data were quantified by sequencing after 24 hours of editing using Illumina NextSeq, circles show each of the three biological replicates, and errors bars are mean ± standard deviation. Additional statistical details are presented in Supplementary Table 3.

bioRxiv preprint doi: https://doi.org/10.1101/2025.06.16.660010; this version posted July 9, 2025. The copyright holder for this preprint (which was not certified by peer review) is the author/funder, who has granted bioRxiv a license to display the preprint in perpetuity. It is made available under aCC-BY 4.0 International license.

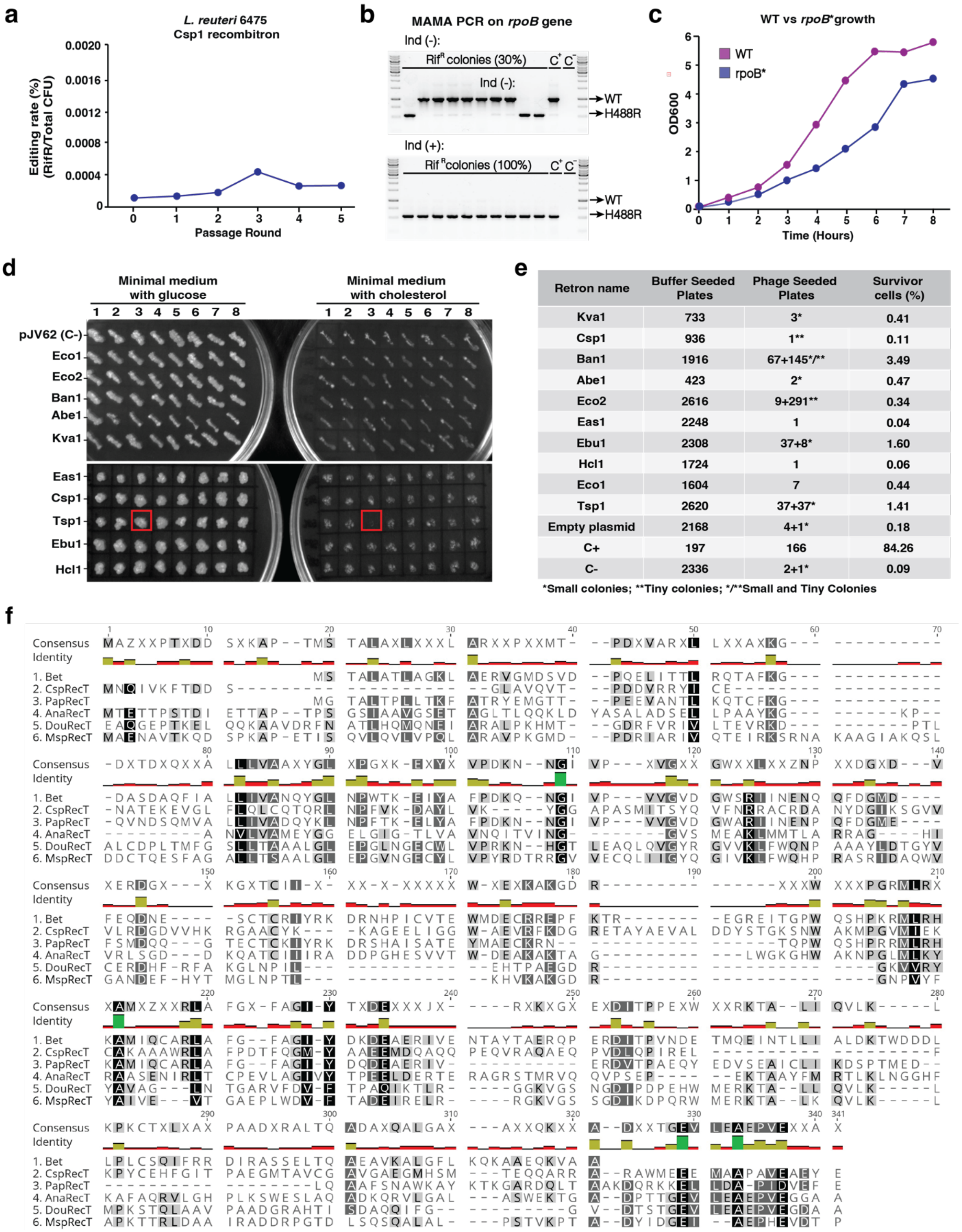

Retron name	Buffer Seeded Plates	Phage Seeded Plates	Survivor cells (%)
Kva1	733	3*	0.41
Csp1	936	1**	0.11
Ban1	1916	67+145*/**	3.49
Abe1	423	2*	0.47
Eco2	2616	9+291**	0.34
Eas1	2248	1	0.04
Ebu1	2308	37+8*	1.60
Hcl1	1724	1	0.06
Eco1	1604	7	0.44
Tsp1	2620	37+37*	1.41
Empty plasmid	2168	4+1*	0.18
C+	197	166	84.26
C-	2336	2+1*	0.09

*Small colonies; **Tiny colonies; */**Small and Tiny Colonies

Supplementary Figure 5 – Retron-mediated recombineering in *Bacillota* and *Actinomycetota*. a, Quantification of precise genome editing to make a single-point mutation in *rpoB* (H488R) gene in *L. reuteri* 6475 using a single plasmid assay with -Csp1 recombitron. The experiment was performed for 6 passage rounds in mMRS with P_{suc} and P_{orfx} inducers. Editing rates were calculated as % of rifampicin resistant colonies respect to total number of colonies in no- antibiotic plates **b**, Mismatch amplification mutation assay (MAMA) PCR to confirm targeted mutations in the *rpoB* gene. Rifampicin-resistant colonies were used as template

bioRxiv preprint doi: https://doi.org/10.1101/2025.06.16.660010; this version posted July 9, 2025. The copyright holder for this preprint (which was not certified by peer review) is the author/funder, who has granted bioRxiv a license to display the preprint in perpetuity. It is made available under aCC-BY 4.0 International license.

for the PCR. Top: screen of 10 colonies with no recombitron induction. Bottom: screen of colonies with recombitron induction. Wild type (WT) and mutant (H488R) bands are indicated with an arrow. **c**, Time course of the OD_{600} of wild type versus mutated *rpoB* gene (H488R) cells. **d**, Minimal Media (MM) plates with glucose or cholesterol with 8 individual colonies of each one of the 10 recombitrons and a negative control (pJV62). No growth of colony 3 on MM with cholesterol indicates that cell was edited by -Tsp1 recombitron. **e**, Quantification of genome editing by infecting with Mycobacteriophage Brilliant. Edited cells are resistant to phage infection. The number of total colonies in the presence of buffer or the phage is indicated. The editing is represented by the percentage of survivor colonies in the phage-seeded plates respect to total colonies in the buffer-seeded plates. **f**, MAFFT Sequence alignment of 6 phylogenetically distinct RecT proteins, including AnaRecT and DouRecT used in *C. acnes* experiments. Sequence shading shows residue conservation and similarity: white text on black background, 80% conserved; grey background, 60% conserved. The alignment was performed using Geneious Prime software.
